# All-Plastic Electrochemical Transistor for Glucose Sensing Using a Ferrocene Mediator

**DOI:** 10.3390/s91209896

**Published:** 2009-12-04

**Authors:** Na Young Shim, Daniel A. Bernards, Daniel J. Macaya, John A. DeFranco, Maria Nikolou, Róisín M. Owens, George G. Malliaras

**Affiliations:** 1 Materials Science and Engineering, Cornell University, Ithaca, NY 14853, USA; 2 Biomedical Engineering, Cornell University, Ithaca, NY 14853, USA; 3 Centre Microélectronique de Provence, Ecole Nationale Supérieure des Mines de Saint Etienne, 13541 Gardanne, France

**Keywords:** organic electrochemical transistor, glucose sensor, ferrocene mediator

## Abstract

We demonstrate a glucose sensor based on an organic electrochemical transistor (OECT) in which the channel, source, drain, and gate electrodes are made from the conducting polymer poly(3,4-ethylenedioxythiophene) doped with poly(styrene sulfonate) (PEDOT:PSS). The OECT employs a ferrocene mediator to shuttle electrons between the enzyme glucose oxidase and a PEDOT:PSS gate electrode. The device can be fabricated using a one-layer patterning process and offers glucose detection down to the micromolar range, consistent with levels present in human saliva.

During the past two decades organic semiconductors have attracted a great deal of attention due to potential applications in a variety of mechanically-flexible, low-cost electronic technologies [1]. A recent trend in the field involves the use of organic semiconductor devices in sensor applications [2,3]. Of particular interest in this arena are organic electrochemical transistors (OECT, also known as conducting polymer transistors). First reported by Wrighton *et al.* in the eighties [4], these devices have been used for the detection of a wide variety of chemical and biological analytes [5,6]. Their mechanism of operation involves modulation of the current that flows in a conducting polymer channel via electrochemical doping or de-doping from ions made available by an electrolyte [7]. OECTs operate at low voltages, which makes them compatible with detection in aqueous environments, and can be miniaturized and integrated with microfluidic channels in a straightforward manner [8], which makes them promising candidates for system-on-a-chip applications. Poly(3,4-ethylenedioxythiophene) doped with poly(styrene sulfonate) (PEDOT:PSS) has emerged as the conducting polymer of choice in OECTs. This is because PEDOT:PSS is commercially available, can be processed into thin films from solution, yields films that are stable in a wide pH range, and has a high conductivity that allows one to fabricate not only the channel, but also the source, drain, and gate electrodes from the same material [9,10].

Over 20 million people are estimated to suffer from diabetes mellitus in the U.S.A. alone, with an estimated cost in excess of one hundred seventy billion dollars in 2007 [11], making glucose sensors a topic of keen interest. Although glucose monitors with adequate capabilities for measuring glucose in blood are commercially available (in people with diabetes the range can be mM [12]), such finger stick assays are painful and costly and as a result participation is limited. Non-invasive measurement of glucose in saliva requires considerably lower detection limits (typical range 0.008–0.21 mM [13]) and necessitates further research and development of sensors. Commonly found glucose sensors employ electrochemical detection utilizing a working electrode (usually Pt) coated with a gel containing glucose oxidase (GOx) [12]. These sensors measure the amount of hydrogen peroxide produced by the reaction cycle seen in Figure 1a [12], according to which glucose is converted to gluconolactone, while the enzyme is reduced and cycles back by producing peroxide. The oxidation of the latter is catalyzed at the Pt electrode and the current measured is proportional to the glucose concentration. Recently, Zhu *et al.* [14] wired a Pt electrode as the gate in a PEDOT:PSS OECT and demonstrated a simple, yet powerful architecture for glucose sensing. In these devices, the change in the potential drop at the Pt/electrolyte interface caused by the reaction cycle in Fig. 1a is accompanied by a change in the gating of the channel in a manner that allows for the determination of glucose concentration [15,16]. However, the Pt gate electrode in these OECTs complicates device fabrication and increases cost, and it is highly desirable to replace it with a PEDOT:PSS electrode.

Electrochemical glucose sensors often replace the O_2_/H_2_O_2_ couple with a fast redox couple, such as the ferrocene [bis (n^5^-cyclopentandienyl) iron] (Fc)/ferricenium ion couple, in order to overcome issues associated with consumption of oxygen [17]. This redox couple shuttles electrons from the reduced enzyme to the working electrode, creating a new pathway as shown in Figure 1b. Given its low redox potential, ferrocene can unload electrons to a PEDOT electrode [18], creating the opportunity to fabricate OECT-based sensors that consist entirely of conducting polymer. In this Letter, we demonstrate such an OECT, in which the channel, source, drain and gate electrodes are made from PEDOT:PSS. The OECT can be used to detect glucose down to the micromolar range, compatible with levels present in human saliva, and can be fabricated using a one-layer patterning process.

Figures 1c–d shows a diagram of the OECT fabrication process and the layout of a finished device. The devices were fabricated on glass slides (7.5 cm × 2.5 cm), though the fabrication process is compatible with most plastic substrates. PEDOT:PSS was patterned using a parylene lift-off technique developed by DeFranco *et al.* [19]. In short, parylene was deposited on the glass slides by chemical vapor deposition to form a 2 μm thick film. Photoresist was spun on top of the parylene, and a contact aligner was used to expose it and define the device pattern. The photoresist was developed and the parylene film was etched using an O_2_ plasma, removing it completely from the substrate in the patterned areas. 20 mL of PEDOT:PSS aqueous dispersion (Baytron P from H.C. Stark) was mixed with 5 mL of pure ethylene glycol and 50 μL of pure dodecyl benzene sulfonic acid (DBSA) and the resulting dispersion was spin coated on the substrates at 1,500 rpm, in order to form a 100 nm thick film. The parylene was peeled off from the substrate leaving only two patterned PEDOT:PSS stripes on top of the substrate. One of these stripes was 0.1 mm wide and was used as the channel (with its outer edges used as source and drain electrodes), while the other was 1mm wide and was used as the gate electrode. The distance between the two stripes was 5 mm. The devices were subsequently baked at 140 °C under vacuum for 1 hour and were immersed in deionized water to remove any excess DBSA.

To accommodate the analyte solution, a well made from the silicone elastomer poly(dimethyl-siloxane) (PDMS) was fabricated as described previously [15] and attached on the glass slide as in Figure 1d, defining an active device area of 10 × 10 mm^2^ (the channel and gate areas were 0.1 × 10 mm^2^ and 1 × 10 mm^2^, respectively). The well was preloaded with a mixture consisting of 80 μL of phosphate buffered saline (PBS), 10 μL of glucose oxidase in PBS (500 units/mL), and, for some of the experiments described here, 10 μL of 10 mM ferrocene in ethanol (higher concentrations would lead to ferrocene precipitation). Subsequently, 10 μL of a glucose solution in PBS with concentration from 1 μM to 100 mM was added to the well (therefore, glucose concentration in this paper refers to the concentration of this solution, rather than the concentration of glucose in the well) and the transistor output was measured using two Keithley 2400 SourceMeters controlled by Labview software. The enzyme, the ferrocene and the glucose were obtained from Sigma-Aldrich.

Figure 2 shows the response of an OECT for V_d_ = −0.2 V and for V_g_ pulsed between 0.1 and 0.4 V. The well was preloaded with a mixture that contained ferrocene, as described above, into which 10 μL of 10 mM glucose solution was added. The application of a positive gate voltage causes a reversible decrease in the current that flows in the channel, and the magnitude of the current is associated with the amount of glucose present in the well [14]. The decrease in current is due to de-doping of the polymer channel caused by cations from the electrolyte. The exact amount of de-doping depends on the potential drop between the electrolyte and the polymer channel, which, in turn, depends on charge transfer reactions that take place at the gate electrode. A quantitative treatment of this was recently presented by Bernards *et al.*[16].

Figure 3 shows the normalized response (NR) of an OECT with a well preloaded with a mixture that did (open circles) and did not (open squares) contain ferrocene, as a function of glucose concentration. The data were acquired for V_d_ = −0.2 V and V_g_ = 0.2 V and normalization was done relative to the zero-concentration limit as:

(1) $$NR=\left|\frac{I_{d}^{\text{conc}}-I_{d}^{\text{conc}=0}}{I_{d}^{\text{conc}=0}}\right|$$

where I_d_^conc=0^ is measured for a reference solution without glucose and I_d_^conc^ is measured for a glucose solution at the concentration of interest. This normalization provides a maximum range of response from zero (no glucose) to one (fully de-doped channel) and facilitates comparison between different devices (the typical batch-to-batch reproducibility in terms of I_d_^conc=0^ was of the order of 10%). When the transistor well is preloaded with a mixture that does not contain ferrocene, the normalized response (open squares in Figure 3) is small and shows only a small variation across the glucose concentration range. This is consistent with our previous work in which it was necessary to use Pt electrodes to obtain a high current modulation [14,16].

In contrast, when the transistor well is preloaded with a mixture that contains ferrocene, the normalized response (open circles in Figure 3) increases dramatically across the glucose concentration range. Namely, NR starts at 0.13 for the 1 μM solution and increases to 0.57 for the 100 mM one. Adequate change in NR is observed in the 1–200 μM range, which is relevant for detection of glucose in human saliva. The results are consistent with the reaction cycle shown in Figure 1b, according to which the ferrocene/ferrocenium ion couple mediates electron transfer between the redox enzyme and the PEDOT gate. In agreement with the model by Bernards *et al.* [16], the flow of electrons to the gate electrode decreases the potential drop at the gate/electrolyte interface. As the gate electrode is held at a fixed bias with respect to the channel, the potential drop at the electrolyte/channel interface increases. The latter results in more effective gating of the transistor channel and the drain current decreases in a way that depends on glucose concentration.

## Conclusions

In conclusion, by employing a ferrocene mediator to shuttle electrons between the enzyme glucose oxidase and a PEDOT:PSS gate electrode, we have demonstrated a glucose sensor based on an organic electrochemical transistor in which the channel, source, drain, and gate electrodes are made from PEDOT:PSS. The device offers a simple architecture for enzymatic sensing that can be fabricated using a one-layer patterning process. Despite its simplicity, it offers glucose detection down to the micromolar range, consistent with levels present in human saliva. Coupled with appropriate enzymes, this OECT architecture might allow the detection of additional metabolites.

## Figures and Tables

**Figure 1.**
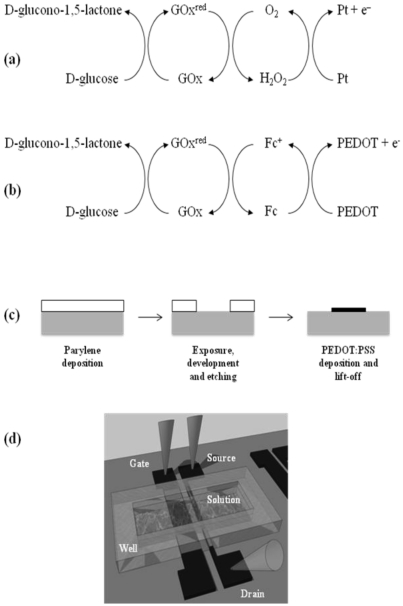
Reaction cycles for detection of glucose in devices utilizing a Pt electrode (a) and in devices utilizing a PEDOT:PSS electrode and a ferrocene mediator (b). Diagram of the OECT fabrication process (c), and layout of a finished device, not to scale (d).

**Figure 2.**
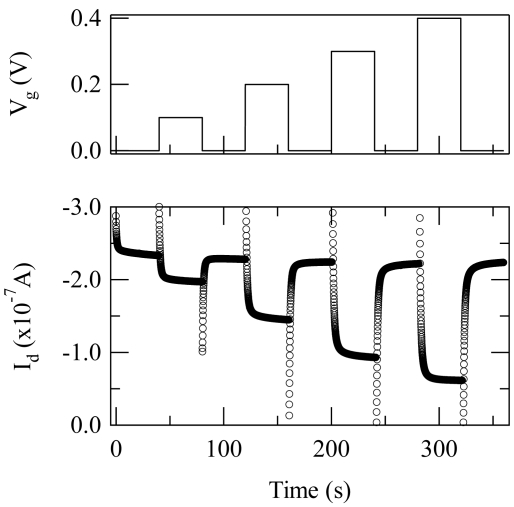
The application of a gate bias (a) causes a modulation of the drain current *I_d_* in a PEDOT:PSS OECT (b).

**Figure 3.**
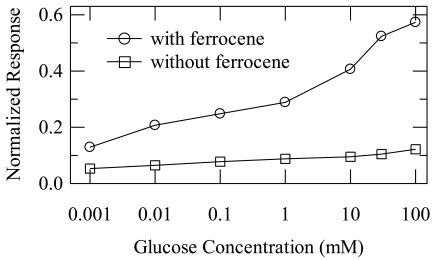
Normalized response to glucose concentration for OECTs preloaded with a mixture with (open circles) and without (open squares) ferrocene mediator. The lines are guides to the eye.
